# Significant Neutrophilic Emperipolesis in Squamous Cell Carcinoma

**DOI:** 10.1155/2018/1301562

**Published:** 2018-11-13

**Authors:** Seza Tetikkurt, Faruk Taş, Funda Emre, Şule Özsoy, Zeki Tolga Bilece

**Affiliations:** ^1^Pathology Department, Bağcılar Training and Research Hospital, University of Health Sciences, Istanbul, Turkey; ^2^Istanbul Medical Faculty, Oncology Institute, Istanbul University, Istanbul, Turkey; ^3^Department of Ear, Nose, and Throat Disease, Bakırköy Sadi Konuk Training and Research Hospital, University of Health Sciences, Istanbul, Turkey

## Abstract

A 53-year-old man was admitted for tooth mobility. A mass was identified at the tooth base by CT. Histopathology of the excisional biopsy revealed a moderately differentiated squamous cell carcinoma. Many intact neutrophils were observed within the malignant cell cytoplasm. The patient underwent partial maxillectomy and bilateral neck dissection. Significant neutrophilic emperipolesis was detected in the resected material. Four tumor recurrences developed in the head and neck region during follow-up. Surgery and chemoradiotherapy was performed. The latest tumor recurrence occurred in the peripharyngeal and the posterior parotideal region. The patient was started on pembrolizumab therapy and nearly complete treatment response occurred. Pembrolizumab was discontinued due to the adrenal insufficiency and pulmonary tuberculosis that developed as a treatment side effect. Pembrolizumab was commenced again when tumor recurrence occurred. The patient is currently alive with ongoing pembrolizumab and antituberculous treatment. We present this case to remark the presence of a significant neutrophilic emperipolesis in the squamous cell carcinoma of the hard palate and maxilla which is rarely encountered. Emperipolesis may predict tumor behavior and the consequences of immune-modulating treatment response in squamous cell carcinomas of the head and neck in regard to the findings of our case.

## 1. Introduction

Emperipolesis is the engulfment of living and intact hematopoietic cells in the cytoplasm of the host cell [1, 2]. The term was first described by Humble et al. in 1956 as “the active penetration of one cell by another which remains intact” [1, 3]. The internalized cells frequently are neutrophils, lymphocytes, and plasma cells. The host cells may be a monocyte, megakaryocyte, endothelial cell, fibroblast, or a malignant cell [1].

Emperipolesis has been observed in various physiological and pathological conditions including cancer and may be the pathognomonic feature of certain diseases [2, 3]. However, it has been so far reported in only one case series that consisted of five patients with oral squamous cell carcinoma [2]. We present a patient with squamous cell carcinoma demonstrating the features of neutrophilic emperipolesis which is a rare pathologic process. Pathologists should be aware that emperipolesis may be observed in squamous cell carcinomas of the head and neck. This finding may a remarkable hallmark for treatment response and prognostic outcome.

## 2. Case Report

**Figure 1:**
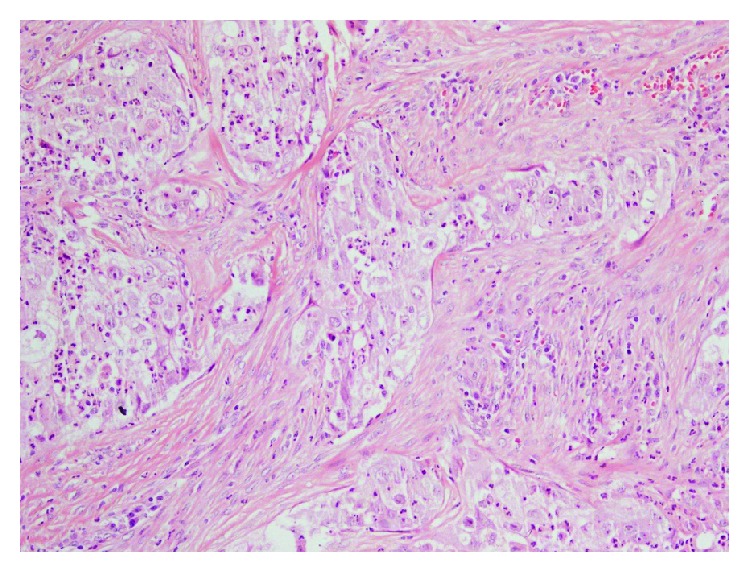
Significant neutrophilic engulfment in the cytoplasm of tumor cells with moderate pleomorphism (H.E., ×200).

**Figure 2:**
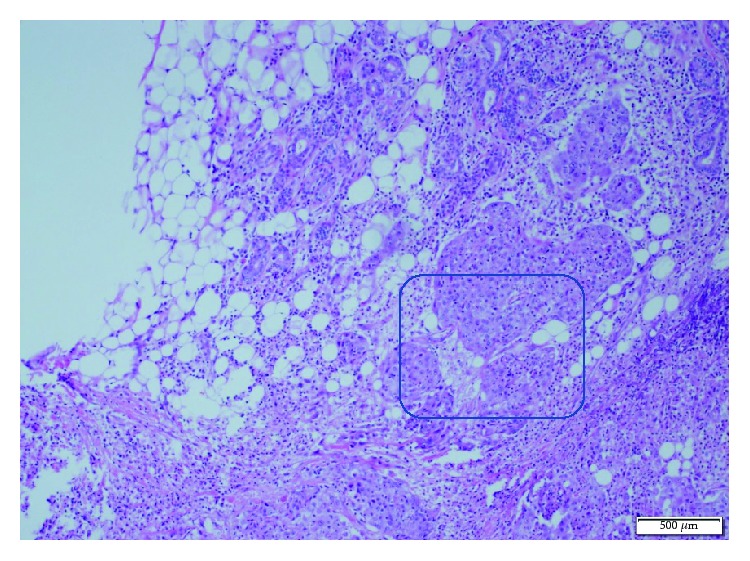
Irregular tumor infiltration to the salivary gland in the resected sample. Significant diffuse neutrophilic infiltration in the tumor cells and surrounding stroma (H.E., ×100).

**Figure 3:**
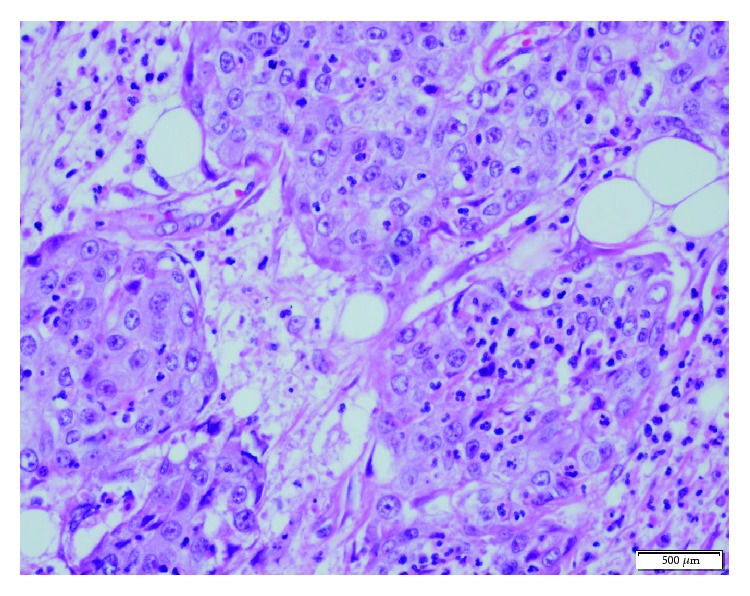
High-power field view of the marked area in Figure 2 (H.E., ×400).

**Figure 4:**
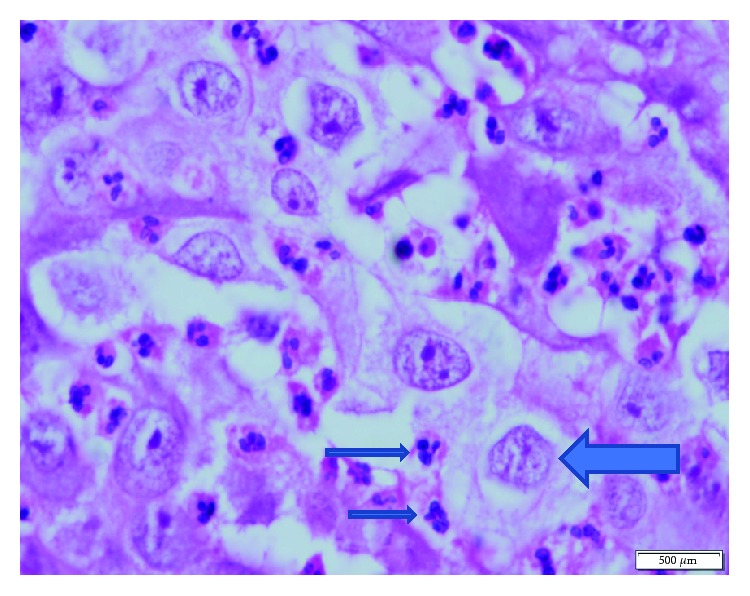
Neutrophil aggregates in the cytoplasm of the tumor cells. Degenerative manifestations were evident and noticeable in some neutrophils. Nucleus of the tumor cell (big arrow) and nucleus of the engulfed neutrophils (small arrows) (H.E., ×1000).

**Figure 5:**
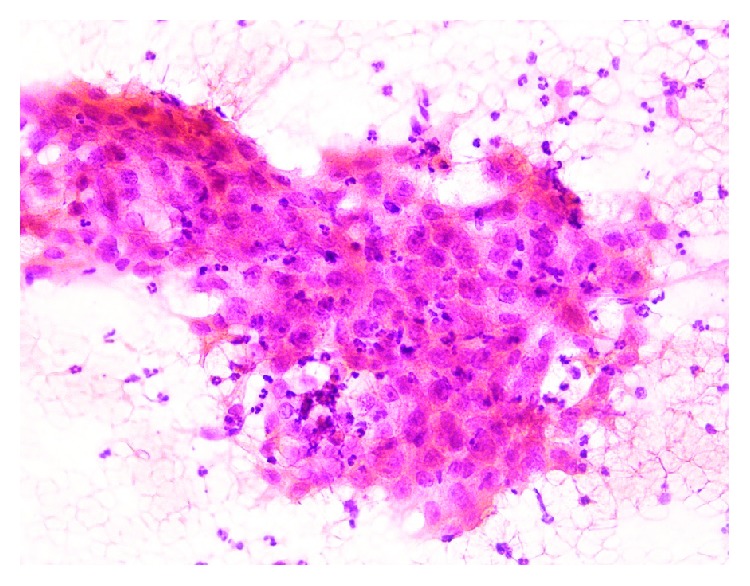
Significant neutrophilic infiltration into the tumor cells and the background in the lymph node aspiration biopsy (PAP, ×400).

**Figure 6:**
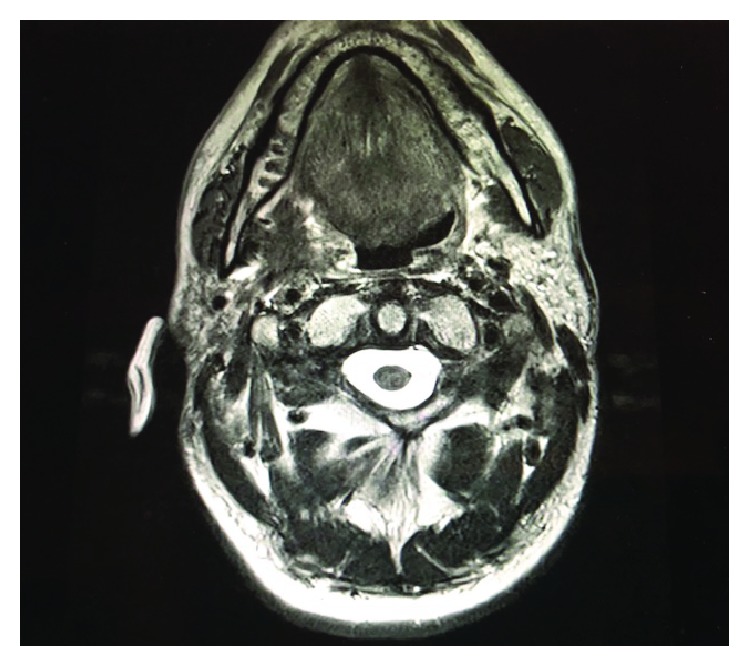
MR image of the solid tumor before pembrolizumab treatment.

**Figure 7:**
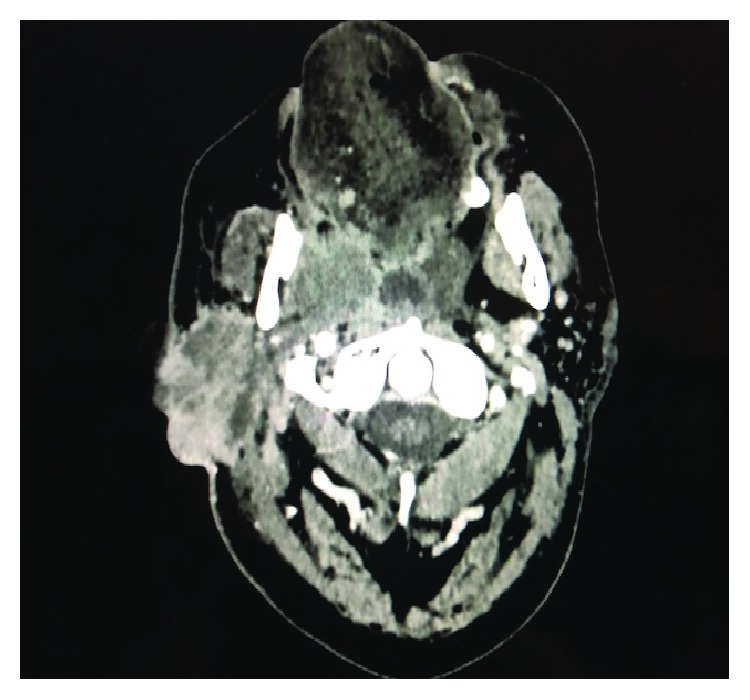
CT image of the rapidly growing tumor when the treatment was first interrupted.

**Figure 8:**
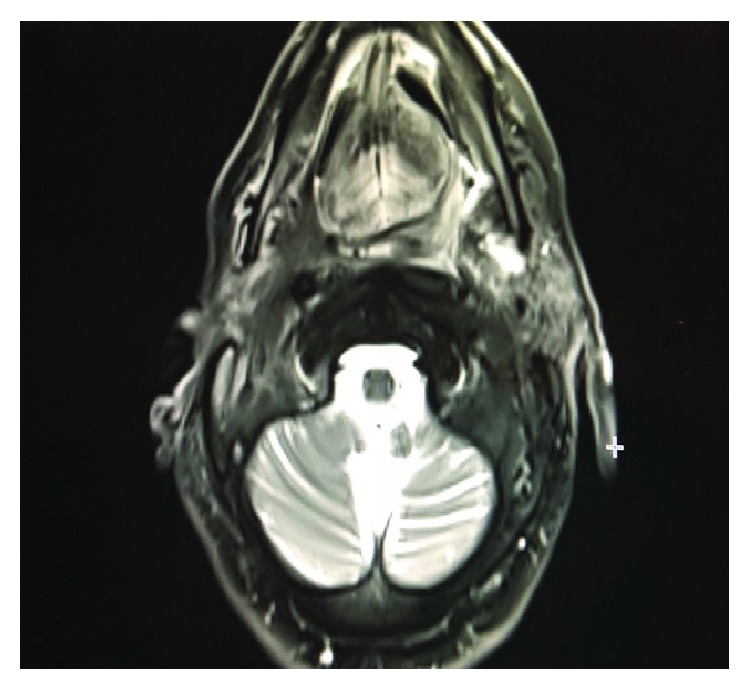
MR image of the tumor following pembrolizumab treatment: decreased tumor size revealing remission.

A 53-year-old man was admitted for mobility of tooth. The dentist suggested the presence of a mass located at the tooth root by physical examination. Computed tomography revealed a well-demarcated radicular cyst of 4 cm in diameter at the tooth base. Carcinomatous infiltration of squamous cell carcinoma was observed in the excisional biopsy of the lesion. In microscopic evaluation, tumoral infiltration revealed features of moderately differentiated squamous cell carcinoma. Malignant cells infiltrated the underlying connective tissue stroma in solid groups and sheets. Numerous neutrophils were present within the cytoplasm of the malignant cells as well as in the surrounding stroma. The internalized neutrophils were intact (Figure 1). Desmosomal connections were observed between the tumor cells in some areas. The tumor cells showed moderate cellular pleomorphism. The diffuse immunoreactivities of P63 and CK5/6 were determined in the malignant cells by immunohistochemical staining. Additionally, perineural invasion was found, whereas vascular invasion was not observed. Because surgical margin was positive for tumor cells, partial maxillectomy and bilateral neck dissection was performed. Bone infiltration was present. Furthermore, diffuse neutrophilic emperipolesis was observed in cancer cells by microscopic evaluation. Some of the neutrophils in the tumor cells revealed degenerative changes by high-power field (×1000) microscopic evaluation (Figures 2–4), while some neutrophils included apoptotic bodies. Nearly one year later, local relapse developed and additional therapeutic manipulations including surgery, radiotherapy, and chemotherapy (cisplatin) were done. Tumor recurrence occurred in the periparotid and right neck lymph nodes after six months (Figure 5). Chemotherapy (cisplatin) and radiotherapy were performed for the recurrence. Two more relapses developed in the right neck, left submandibular lymph nodes and in the superficial and deep soft tissues of the neck three months apart. Tumor showed continuity along the surgical margin in the excised biopsy sample and a pericapsular invasion at the submandibular lymph node. Chemotherapy was continued. The endmost tumor recurrence was in the palatine tonsil and posterior parotideal region. Following unresponsive chemotherapy, pembrolizumab treatment was started eight months prior to this study (Figure 6). A complete response occurred following the sixth dose of pembrolizumab. Secondary adrenal insufficiency and pulmonary reactivation tuberculosis developed as the side effects of treatment. Tuberculosis was identified by PCR and compatible chest CT findings. Pembrolizumab was interrupted and antituberculous treatment was started. Pembrolizumab was commenced when the tumor progressed to a 15 cm mass (Figure 7). Following the fourth dose of pembrolizumab, the tumor regressed to 4 cm (Figure 8) and the patient is currently alive for four years.

## 3. Discussion

It is estimated that about 51,540 new cases of oral cavity and pharyngeal cancers will occur that account for about 4% of the new cancer cases in the US in 2018. This will result in an estimated 10,030 deaths from head and neck cancers during the same time period. Squamous cell carcinoma is the histologic type in more than 90% of these tumors. Because approximately 70% of the cases present with advanced disease, only 65% of the patients will be alive 5 years after diagnosis [4].

Squamous cell carcinoma is usually preceded by premalignant disorders, whereas a detailed tumor biology of this process is unknown. Until today, emperipolesis was investigated in oral squamous cell carcinoma patients only in one report [2]. In the analysis of 56 squamous cell carcinoma cases, the features of this process were observed in only five patients. Both partial and complete engulfment of lymphocytes by tumor cells were present in these cases. There were no signs of degeneration appreciable in either cell, thus ruling out the possibility of the phenomenon of cannibalism [2]. In our case, tumor cells revealed abundant neutrophils in their cytoplasm and in the surrounding stroma. In addition, some neutrophils revealed degenerative changes and occasional apoptotic bodies in the highest power field microscopy (×1000). Moreover Sarode and Sarode reported seven cases of oral squamous cell carcinomas that showed neutrophil-tumor cell cannibalism among 500 patients. The neutrophils in the tumor cell cytoplasm revealed different stages of degeneration in this study [5]. Neutrophil-tumor cell emperipolesis or phagocytosis (cannibalism) has been identified by light microscopy evaluation of pleomorphic cell (giant cell) carcinomas of the intestine, lung, and pancreas, breast carcinomas, invasive micropapillary carcinomas of the ampulopancreaticobiliary region, gastric carcinomas, and lymphomas [6–12]. Some cases of gastric carcinoma revealed apoptotic neutrophils within the cytoplasmic vacuoles of adenocarcinoma cells that were observed by an ultrastructural study [12].

Phagocytic behavior has been reported in many types of cancer. In these studies, phagocytic/cannibalistic behavior was confined to the highly invasive and metastatic cells. [13–15]. Cannibalism has been described in bladder, breast, and lung cancers and is related with the aggressiveness of the malignancy [16]. Phagocytosis is a specific type of behavior for M2 macrophages and other phagocytes. M2 macrophages may also produce high levels of lysosomal-enriched cathepsins. The high concentration of these enzymes in breast, brain, colon, or endometrium tumors was denoted as a sign for high malignancy, aggressive metastatic potential, and overall poor prognosis [17, 18]. Cell cannibalism and entosis are the other forms of cell-in-cell phenomena. Entosis is described as a cell that engulfs another cell of the same type. The cells that are internalized by this mechanism are initially viable with the majority of them eventually undergoing a nonapoptotic form of cell death that requires autophagy proteins. In cannibalism, the engulfed cell still remains alive when internalized but the process implies its death. The tumor cells are seen engulfing other tumor cells, neutrophils, and erythrocytes [1].

Neutrophils may lead to a significant impact on the tumor microenvironment through cytokines and chemokines that cause inflammatory cell recruitment and activation. Many cells in the tumor microenvironment may secrete neutrophil chemotactic factors. The specific role of neutrophils and macrophages in the pathogenesis of cancer has recently become the subject of research with special focus on the association between inflammation and cancer progression. Neutrophils may play a crucial role in cancer biology and different subpopulations may act as antitumor or protumor in cancer progression. Neutrophils may be important biomarkers for squamous cell carcinoma and targets to control cancer progression [19, 20].

Recurrent head and neck squamous cell carcinomas (HNSCCs) are a treatment challenge for the clinician because of the effects of prior treatment, multifocality with an infiltrative nature, and a propensity for tumor recurrence [21]. Invasion pattern, neoadjuvant chemotherapy, surgical margin status, and extracapsular spread of lymph nodes were identified as factors associated with the local recurrence of oral squamous cell carcinomas [22, 23]. Bone invasion, positive surgical margins, and pericapsular invasion of the cervical lymph node appear to be the fundamental factors that becloud the local tumor control in our case. The hallmark for the recurrence of the tumors in the neck region may be the presence of tumor invasion at the surgical margins.

The significance of emperipolesis in neoplastic disorders is still obscure. The neutrophil-tumor cell emperipolesis, entosis, and/or phagocytosis (cannibalism) may be a spectrum of the cell-in-cell phenomenon. This may be a permanent and successive process or a focal and a limited condition of cell death in some cases. Distinctive features to differentiate emperipolesis from other forms of the cell-in-cell phenomenon, such as cannibalism and entosis, in squamous cell carcinoma patient studies with larger sample sizes are needed for the determination of its role in the clinicopathological and therapeutic mechanisms. Currently, the underlying pathogenetic mechanism is unclear. Emperipolesis may be a predictor for tumoral process/progression and immune-modulating therapy response for the squamous cell carcinomas of the head and neck.
